# The Roles of Monocytes and Macrophages in Behçet’s Disease With Focus on M1 and M2 Polarization

**DOI:** 10.3389/fimmu.2022.852297

**Published:** 2022-03-11

**Authors:** Lisa Hirahara, Kaoru Takase-Minegishi, Yohei Kirino, Yuki Iizuka-Iribe, Yutaro Soejima, Ryusuke Yoshimi, Hideaki Nakajima

**Affiliations:** Department of Stem Cell and Immune Regulation, Yokohama City University Graduate School of Medicine, Yokohama, Japan

**Keywords:** polarization, genetics, innate immunity, macrophages, monocytes, Behcet’s disease

## Abstract

Behçet’s disease (BD) is a systemic inflammatory disease characterized by recurrent oral ulcers, genital ulcers, cutaneous inflammation, and uveitis. In addition, other potentially life-threatening lesions may occur in the intestinal tract, blood vessels, and central nervous system. This heterogeneity of the BD phenotype hampers development of a targeted treatment strategy. The pathogenesis of BD is not fully elucidated, but it is likely that genetically susceptible people develop BD in response to environmental factors, such as microbiome factors. Genetic analyses have identified various BD susceptibility loci that function in HLA-antigen presentation pathways, Th1 and Th17 cells, and autoinflammation related to monocytes/macrophages, or that increase levels of pro-inflammatory cytokines, reduce levels of anti-inflammatory cytokines, or act in dysfunctional mucous barriers. Our functional analyses have revealed that impairment of M2 monocyte/macrophage-mediated anti-inflammatory function through IL-10 is crucial to BD pathogenesis. We, therefore, propose that BD is an M1-dominant disease. In this review, we describe the roles of monocytes and macrophages in BD and consider the potential of these cells as therapeutic targets.

## Introduction

Behçet’s disease (BD) is a systemic inflammatory disease initially reported in 1937 by Hulusi Behçet, a prominent Turkish dermatologist. The disease is epidemiologically characterized by a high incidence along the ancient Silk Route, and Professor Shigeaki Ohno has named the disease “Silk Road Disease” (1). The typical manifestations of BD are recurrent oral ulcers, genital ulcers, cutaneous inflammation, and uveitis. However, other potentially life-threatening lesions may occur in the intestinal tract, blood vessels, and central nervous system and this heterogeneity results in the disease being considered a syndrome (2). There are currently no disease-specific antibodies or biomarkers for BD; therefore, diagnosis is made solely on clinical symptoms. The most important symptom is recurrent oral ulcers, which is seen in more than 95% of Japanese patients and is mandatory for diagnosis according to the International Study Group criteria (3, 4). The pathogenesis of BD is not fully determined, but it is likely that people who are genetically susceptible to the disease may develop BD as a response to environmental factors, such as microbiome factors. HLA-B*51 is the most widely known BD susceptibility gene, with an odds ratio of 5.9 for the development of BD, but its allele frequency is about 20% in the Japanese population and is, therefore, not a disease-causative locus (5). Genetic analyses have identified various susceptibility loci involved in HLA-antigen presentation pathways, Th1 and Th17 cells, pro-inflammatory cytokine regulation, dysfunctional mucous barriers, and autoinflammation related to monocytes/macrophages, the main focus of this review. Our research goal is to categorize the complexity of BD and to establish treatment strategies that are tailored to individual patients. In this respect, macrophages and monocytes could be important targets. In this review, we discuss the roles of monocytes and macrophages based on the results of a recent literature survey and how monocyte-macrophages may be considered as therapeutic targets of BD.

## Elucidation of Monocyte Function

### Neutrophil Hyperchemotaxis and Monocytes in BD

Early studies of the involvement of monocytes and macrophages in BD focused on their function as part of the pathogenesis of enhanced neutrophil chemotaxis in response to environmental factors. Aberrant neutrophil chemotaxis in BD was reported in 1975 and colchicine was found to exert a therapeutic effect by inhibiting neutrophil migration (6). Monocyte involvement in the mechanism of neutrophil chemotaxis was then investigated. In a report from 1993, monocytes from patients with active BD displayed increased secretion of pro-inflammatory cytokines, including TNF-α, IL-6, and IL-8 (7). Then, in 1995, increased levels of the soluble monocyte-activation marker, CD14, were reported in the sera of BD patients, and monocyte culture supernatants from BD patients were shown to significantly enhance neutrophil adhesion to endothelial cells (8). These results indicated that monocytes in BD patients are highly activated and involved in chronic inflammation by continuous production of pro-inflammatory cytokines. Histopathological examination then revealed that the cells infiltrating oral ulcer sites in BD patients were lymphocytes and monocytes/macrophages (9). Furthermore, mononuclear cells consisting of CD4^+^ T cells and monocytes were demonstrated to infiltrate the periphery of small blood vessels at the site of the pathergy reaction, suggesting that monocytes are the essential driver of inflammation in BD (10).

### Activation of the Innate Immune System *via* Toll-Like Receptors

Involvement of monocytes in the pathogenesis of BD was further strengthened by the discovery of the pattern recognition mechanism of Toll-like receptors (TLRs). TLRs are a defense mechanism against pathogens-associated molecular patterns (PAMPs) or damage-associated molecular patterns (DAMPs). Microbes have long been assumed to be an external factor triggering BD inflammation. In fact, Hulusi Behçet reported that BD is induced by herpes virus infection, and herpes simplex-induced animal models elicit BD-like symptoms (11). Herpes viruses replicate in monocytes and can be detected by TLR2 and TLR9, which are highly expressed in monocytes.

Oral commensal bacteria are another candidate risk factor for BD. It is hypothesized that streptococci are particularly prevalent in BD because oral ulcers often worsen after dental treatment (12). Heat shock protein (HSP) 65 produced by these bacteria is homologous to human HSP60, and hyper-reactivity of lymphocytes to HSP has been reported in BD patients. Ten human TLRs have been identified and peptidoglycan (PTG) is a ligand for TLR2 and lipopolysaccharide (LPS) is a ligand for TLR4. Furthermore, HSP60 can bind to several PAMPs and induces cytokine production *via* TLR2 and TLR4 signaling, indicating that TLR2 and TLR4 are involved in the pathogenesis of BD (13).

In 2008, we reported the overexpression of TLR4 in peripheral blood mononuclear cells (PBMCs) of BD patients (14). Subsequently, it was reported that TLR2/TLR4 expression was also increased in monocytes from BD patients (15). In 2013, upregulated expression of TLR2/TLR4 was found in macrophages isolated from BD patients, and that TLR2/TLR4-mediated IL-1β was upregulated in patients with active uveitis when stimulated with peptidoglycan/LPS (16). These results indicate that monocytes are involved in BD pathogenesis in part by activating the innate immune system against external stimuli *via* the TLR pathway. Concordant with these observations, a targeted resequencing of innate-immune genes in Japanese and Turkish populations identified low-frequency *TLR4* variants associated with BD, supporting the hypothesis of innate immune system activation through TLRs in BD (17).

## Functions of Monocytes and Macrophages Revealed by Genetic Studies

**Figure 1:**
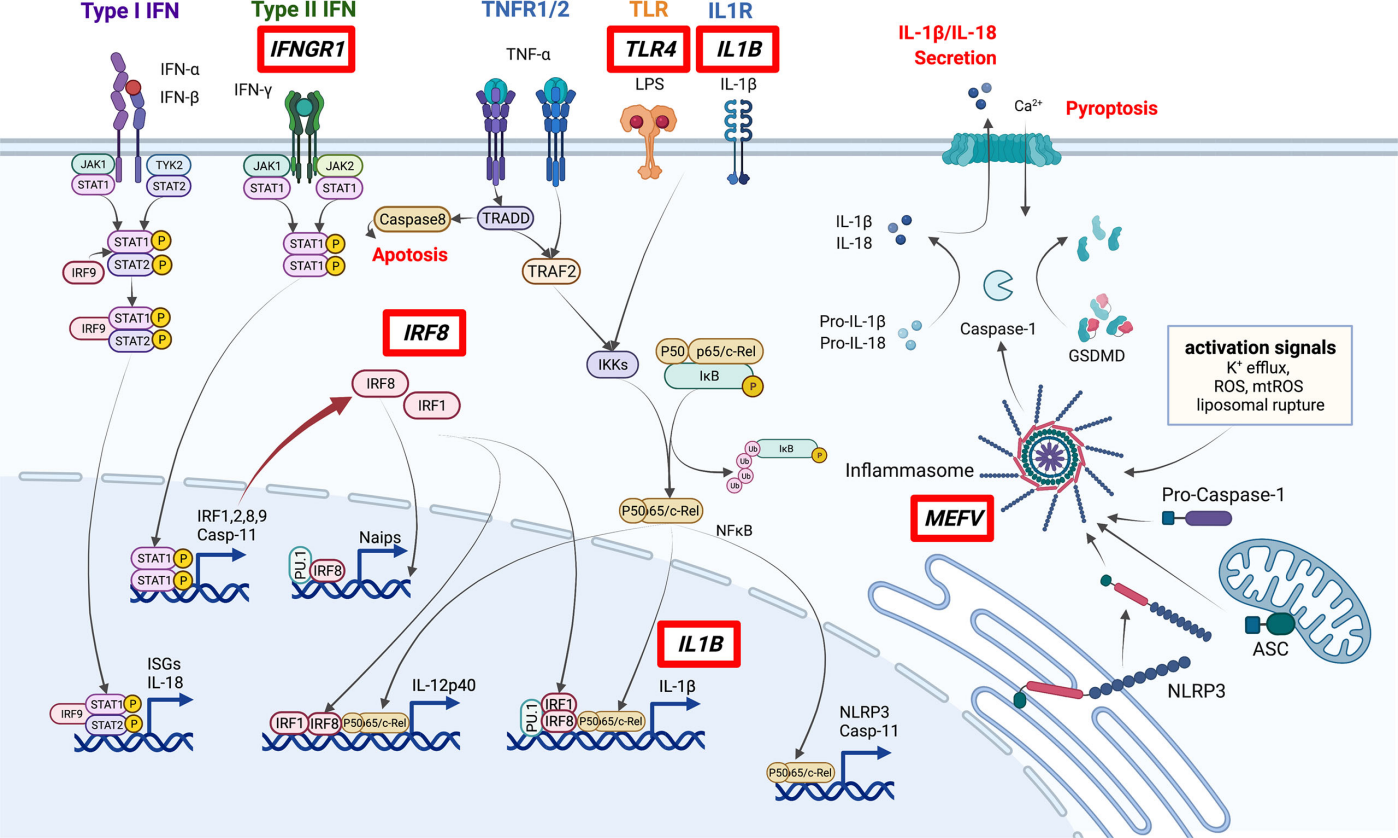
Pathways involved in the pathogenesis of BD. Genes discovered by GWAS to be associated with BD (red boxes) include several involved in macrophage inflammation. In particular, IRF8 and MEFV are important in the regulation of STAT signaling and inflammasome activation. ISGs: Interferon-stimulated genes. This figure was created with BioRender.com.

Since 2010, genome-wide association studies (GWAS) and other large-scale genetics studies have been conducted to explore BD. These investigations confirmed that the HLA region has the highest association with BD development. HLA-B*51, A*26, B*15, B*27, and B*57 were identified as disease-susceptibility alleles, and A*03 and B*49 were identified as disease-protective alleles (18). In addition to HLA genes, nearly 20 disease-susceptibility loci were identified, including *IL10*, *IL23R*, and *ERAP1*, which contributed to the proposal of the disease concept of “MHC class-I-opathy”, similar to spondyloarthritis (**Table 1**) (19–21, 23). BD-related loci that may affect monocyte and macrophage function include *IL10*, *CCR1–CCR3*, *MEFV*, *IL1B*, *IRF8*, and most recently, *IFNGR1* (27, 28). The pathways associated with BD are summarized in **Figure 1**.

**Table 1:** Genome-wide significant disease susceptibility loci for BD.

Year	Author	Population	Gene
**2009**	Meguro et al. (19)	Japanese	*HLA-A26*
**2010**	Mizuki et al. (20)	Japanese	***IL10****, IL23R*
**2010**	Remmers et al. (21)	Turkish, mixed populations	***IL10****, IL23R*
**2012**	Hou et al. (22)	Chinese Han	*STAT4*
**2013**	Kirino et al. (23)	Turkish, Japanese	***CCR1-CCR3****, STAT4, KLRC4, ERAP1*
**2013**	Kirino et al. (17)	Turkish	***MEFV M694V***
**2013**	Lee et al. (24)	Korean, Japanese	*GIMAP*
**2014**	Ombrello et al. (18)	Turkish	*HLA-A03*
**2014**	Xavier et al. (25)	Iranian	*FUT2*
**2015**	Kappen JH et al. (26)	Turkish and mixed populations	*IL12A*
**2017**	Takeuchi et al. (27)	Turkish, Japanese, Iranian	***IL1B***, ***IRF8****, RIPK2*, *EGR2, LACC1, PTPN1*
**2020**	Ortiz Fernández et al. (28)	Turkish, Japanese etc.	***IFNGR1****, DKK1*

The genes highlighted in bold are the ones that we focused on in this paper as being related to macrophage and monocyte function.

### Elucidation and Interpretation of CCR1 Function

An important finding in understanding the function of monocytes and macrophages in the pathogenesis of BD was identification of the *CCR1–CCR3* locus. *CCR1* encodes CC-motif chemokine receptor1(CCR1), which is highly expressed on monocytes/macrophages, whereas CCR3 is highly expressed on eosinophils, basophils, and T cells. The ligands for CCR1 are macrophage inflammatory protein-1 alpha (MIP-1α), regulated on activation normal T expressed and secreted protein (RANTES), and monocyte chemoattractant protein 3 (MCP3). Upon detection of these chemokines, monocytes migrate to the site of high chemokine production (29). GWAS identified a locus tagged with SNP rs7616215 located in the 3′ non-cording region of *CCR1*, and functional analysis revealed that risk allele T was associated with reduced expression of *CCR1*. Furthermore, the migratory ability of monocytes in response to MIP-1α was lower in individuals with risk allele T (23). These results indicate that monocyte chemotaxis is reduced in patients with BD, in contrast to increased monocyte infiltration of lesions. Inconsistent with our GWAS findings, systematic expression quantitative trait analysis with various cell subsets indicated that rs7616215 affects *CCR3* more than *CCR1*, resulting in higher *CCR3* expression (30). However, a recent large GWAS on canker sores, a refractory form of which is considered a “Behçet-spectrum disorder” (31), confirmed association between *CCR1-CCR3*, and the Genotype-Tissue expression of *CCR1* and *CCR3* were down-regulated and up-regulated, respectively, indicating that the locus has a binary effect on *CCR1* and *CCR3* expression (32). In addition, a recent single-cell whole-genome expression quantitative trait analysis (33) showed consistent results that the risk allele T is associated with decreased expression of CCR1 and increased expression of CCR3 in classical monocytes as well as neutrophils and plasmacytoid dendritic cells (**Supplementary Figure 2**).

### Abnormal Function of Polarized Macrophages in the Context of IL10

Macrophage polarization has attracted much attention in recent years. M1 macrophages secrete pro-inflammatory cytokines, such as IL-1, IL-6, and TNF-α, which are therapeutic targets of BD, while M2 macrophages secrete anti-inflammatory cytokine represented by IL-10 (34). M2 can be further classified into four subtypes, M2a, M2b, M2c and M2d. Each subtype is characterized by cell surface markers, secreted cytokines and function, but all subtypes secrete IL-10 (35). IL-10 receptor deficiency is known to cause severe inflammation in skin and intestines, symptoms that partially overlap with BD (36). GWAS showed association of BD with *IL10* intronic variant, rs1518111, in both Japanese and Turkish populations (20, 21). *IL10* mRNA and protein levels were reduced in monocytes of individuals with risk alleles compared with those in individuals without risk alleles (21). As mentioned earlier, *CCR1–CCR3* SNPs are associated with BD, and reduced expression of *CCR1* is associated with disease risk.

We addressed how decreased monocyte migration ability revealed by GWAS is related to the pathogenesis of BD by analyzing the polarization of M1 and M2 macrophages (37). Expression of *IL10* and *CCR1* was generally higher in M2 than M1 macrophages, and *CCR1* expression in M1 macrophages was higher in BD patients than in healthy controls. We also found significant infiltration of M1 macrophages in erythema nodosum lesions of BD patients. *CCR1* SNP, rs7616215, is a BD-risk allele associated with reduced M2 migration in response to MIP-1α. These results suggest that M2 macrophage infiltration capacity is lower in BD than in healthy subjects, and that fewer M2 cells and BD-associated SNPs may result in lower levels of IL-10, resulting in M1-predominant inflammation in BD. As mentioned above, recent quantitative trait analysis of single cell expression revealed that CCR1 has a role in the migration of various immune cells, and that not only monocytes but also neutrophils and acquired immune responses are affected by this CCR1-CCR3 locus (**Supplementary Figure 2**). The crosstalk between polarized macrophages and neutrophils and acquired immune cells is not fully understood, but the interaction may be important for inflammation in BD.

**Figure 2:**
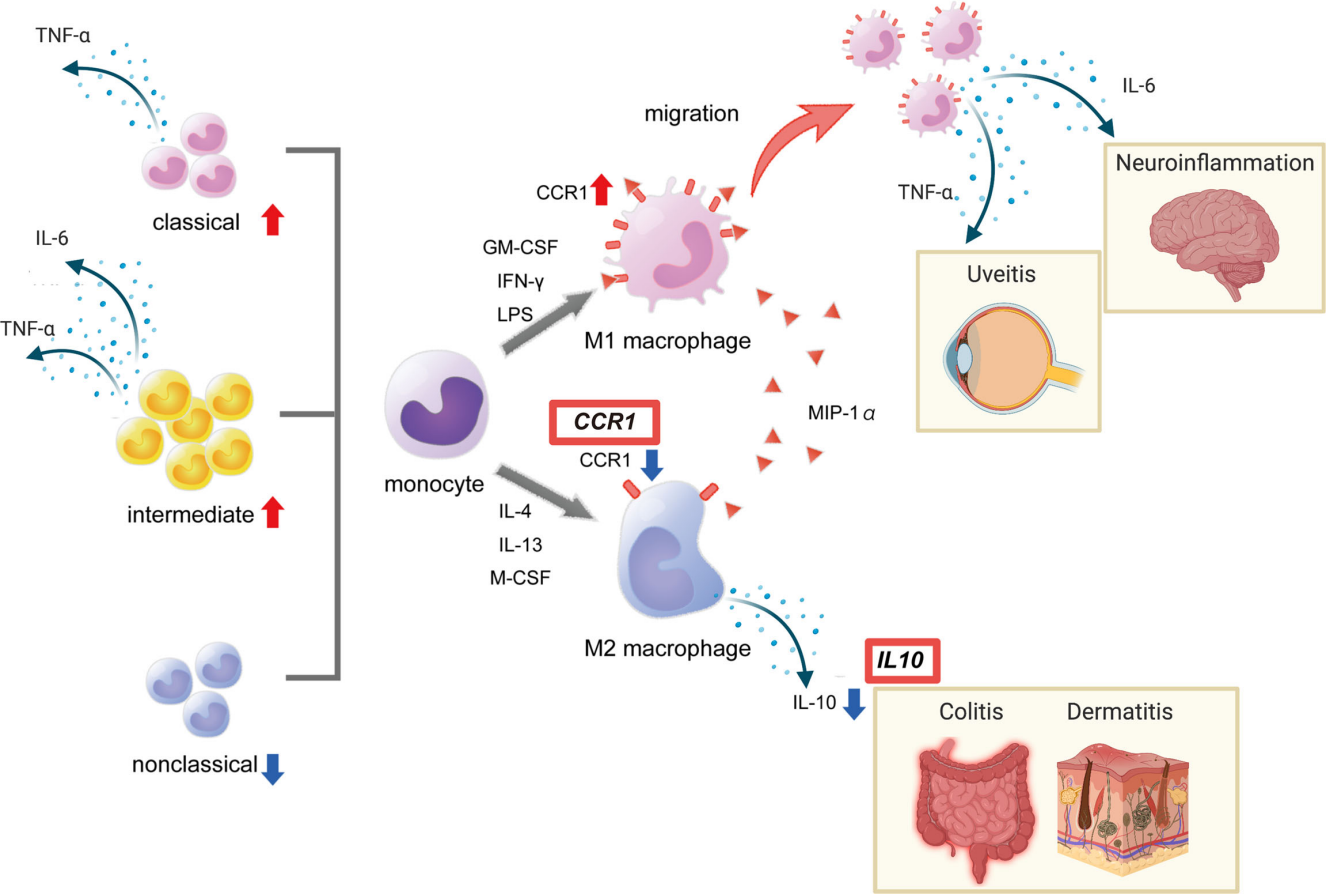
Red boxes show disease susceptibility genes for BD associated with this pathway. This figure was created with BioRender.com.

In line with our findings, macrophages collected from the peritoneal fluid of herpes simplex virus-induced BD model mice were predominantly M1 (38). Macrophages collected from healthy individuals treated with serum from BD patients promoted differentiation into M1 macrophages, while differentiation into M2 macrophages was suppressed (39). We also reported that the production of heme oxygenase 1, an anti-inflammatory heme degrading enzyme, is high in M2 macrophages but reduced in BD patients in response to TLR4 stimulation (14). Together these results support the hypothesis of impaired M2 function in BD (**Figure 2**).

**Figure 3:**
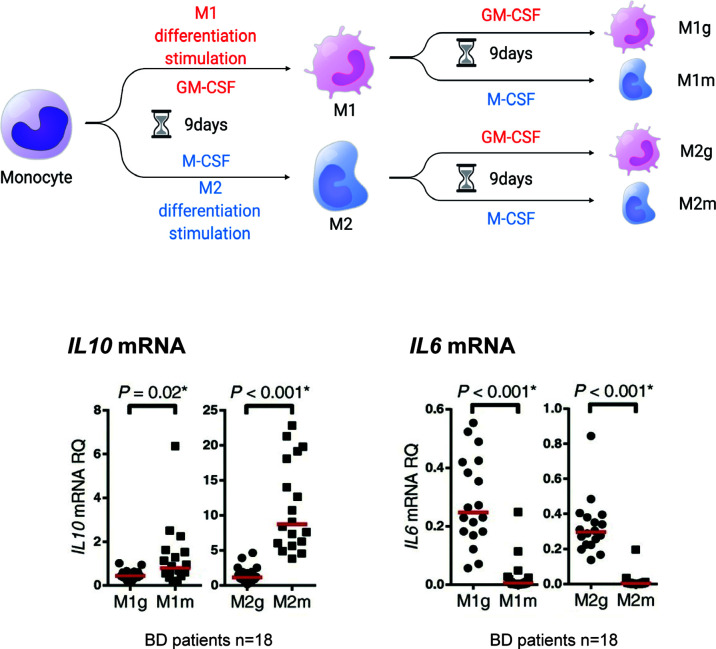
M1 and M2 macrophages may be interchangeable. Monocytes (mono) treated *in vitro* with GM-CSF (M1g and M2g) or M-CSF (M1m and M2m) for 9 days developed an M1 or M2 macrophage-like phenotype (defined here as M1 or M2 stimulation). However, the M2 macrophages stimulated with “M2” or “M1” stimulation for another 9 days (the first M1 stimulus followed by M2 stimulus is defined as “M2g” and the second M1 stimulus followed by M2 stimulus is defined as “M1m”) maintained or decreased *IL10* mRNA production. *IL6* mRNA production was reduced by M2 stimulation of M1 macrophages. This figure is a modified version of Figure 4 a-e published in Nakano et al. (37). The figure is modified and distributed under the Creative Commons Attribution 4.0 International License (CCBY4.0, http://creativecommons.org/licenses/by/4.0/). This figure was partially created by BioRender.com.

Importantly, anti-inflammatory M2 macrophages may not be consistently anti-inflammatory. We previously tested whether established M1 macrophages could be converted to M2 macrophages in human primary cells (37). For this purpose, M1 and M2 macrophages that underwent 9 days of *in vitro* polarization in response to GM-CSF or M-CSF were cultured for another 9 days in the presence of the same or other cytokines. Treatment with M-CSF restored the expression of *IL10* (**Figure 3**). Conversely, the expression of *IL10* mRNA was decreased by GM-CSF in M2 macrophages. *IL6* mRNA also showed changes in this experiment. These results suggest that the macrophage phenotype is partially interchangeable between M1 and M2, depending on situational factors, including cytokines.

## Aberrant Monocyte Subsets in BD Patients

Interestingly, several recent reports have shown that not only macrophages but also monocyte subsets are imbalanced in BD. Based on the expression of molecules on the cell surface, monocytes can be classified into three categories: classical (CD14^++^CD16^-^), intermediate (CD14^++^CD16^+^), and non-classical (CD14^+^CD16^++^) (40). These three subtypes are also considered to be functionally distinct. Classical monocyte represents the majority of circulating monocytes. In general, classical monocytes have a pro-inflammatory and phagocytic phenotype. They express high level of CCR2, migrate in response to CCL2(MCP-1) stimulation and are involved in initiation of inflammation. In contrast, non-classical monocytes that patrol the vascular endothelium to survey for damage. Particularly in the area of vascular diseases, they have been focused on as intravascular scavenger that remove cellular debris from blood vessels (41, 42). They produce less reactive oxygen species (ROS) and cytokines in response to stimulation with cell surface TLRs, but produce pro-inflammatory cytokines in response to stimulation with viruses and immune complexes containing nucleic acids (43). Although the functions of intermediate monocytes are not fully understood, they are involved in ROS production and highly express genes related to antigen presentation, cytokine production, apoptosis control, T cell proliferation and cell differentiation (44, 45).

Increased numbers of classical monocytes and decreased numbers of nonclassical monocytes have been reported in BD patients with uveitis (46). In addition, monocytes from BD patients and healthy controls differ in TLR expression by subset, with classical monocytes expressing TLR2, TLR4, and TLR5, nonclassical monocytes expressing TLR2 and TLR5, and intermediate monocytes expressing TLR2 (47). BD patients have increased numbers of intermediate monocytes and decreased numbers of nonclassical monocytes compared with healthy controls. The results showed that classical and intermediate monocytes overproduce TNF-α and intermediate monocytes overproduce IL-6 (48). At present, little is known about monocyte subsets in BD patients. However, there is also a theory that non-classical monocytes can differentiate into M2 macrophages; hopefully future research will answer these questions.

### IL1A/IL1B and MEFV

Immunochip analysis in Turkish, Japanese, and Iranian subjects revealed association of *IL1A–IL1B* and *IRF8* loci with BD. Functional analysis revealed that risk SNPs in the *IL1A–IL1B* region were associated with high levels of *IL1β* expression (27). Targeted-resequencing of innate-immune genes in a Turkish population identified association of BD with *MEFV M694V*, a known disease-causing variant of familial Mediterranean fever. *MEFV* encodes a pyrin, which forms pyrin-inflammasomes in response to toxins produced by *Clostridium difficile* (17, 49). A pyrin ligand β2-microglobulin (β2MG) induces pyrin inflammasome formation, while the caspase-1p20 subunit produced by the pyrin inflammasome inhibits the pyrin-β2MG interaction in neutrophils (50). The M694V mutation weakens the inhibitory effect of caspase-1p20 on the pyrin-β2MG interaction (50). Inflammasomes are composed of pattern recognition receptors, such as NOD-like receptors, and an adaptor protein, pro-caspase 1 (51). When the inflammasome is formed, pro-caspase 1 becomes activated caspase 1, which cleaves pro-IL-1β into active IL-1β (52). Caspase 1 also cleaves gasdermin D, the N-terminus of which forms membrane pores, leading to pyroptosis (53). This pathway is called the canonical pathway. In addition to this process, there is the noncanonical pathway that releases IL-1β/IL-18 and causes pyroptosis *via* caspase 11 (54). Together, these findings indicate that excessive production of IL-1β by activated inflammasomes is likely to be involved in the pathogenesis of BD.

Conclusions from studies using cells from BD patients have been controversial. In monocyte-derived macrophages from BD patients, TLR2 and TLR4 expression and IL-1β/ROS production were upregulated and IL-1β production was suppressed by inhibition of the NLRP3 inflammasome (55). In PBMCs from BD patients, production of NLRP3 inflammasomes and IL-1β in response to LPS stimulation was increased compared with that in healthy controls (56). The expression of NLRP3, caspase 1 and gasdermin D was also significantly higher in the intestinal tissues of BD patients (57). Meanwhile, no enhancement of the caspase 1 pathway was observed in dendritic cells of BD patients (58). The serum level of gasdermin D was lower in BD patients used as disease controls for adult-onset Still’s disease (59). The differences in these conclusions might be affected by different cell types and patients’ backgrounds. Focusing on the relationship between risk alleles and monocyte/macrophage polarity may provide additional autoinflammatory insights into BD.

### IRF8

IRF8 is one of the nine members of the interferon regulatory factor (IRF) family (60). In IRF8-deficient mice, monocytic phagocyte differentiation is inhibited and abnormal differentiation into neutrophils occurs, indicating that IRF8 regulates the differentiation of progenitor cells into monocytic phagocytes (61, 62). IRF8 is also involved in the formation of M1 macrophages (63).

IRFs also function in signaling by TLRs and IFN receptors. IRF8 is expressed in macrophages and is responsible for induction of interferon production; IRF8 regulates transcription by forming a complex with IRF1 and transcription factors such as AP1 and PU.1 (64–67). Stimulation of myeloid cells with LPS results in increased expression of IRF8, which then binds to sites on the genome that are different from its steady-state binding site (68). The activity of IRF8/PU.1 is required for the regulation of *IL18* expression in macrophages in mice (69). Furthermore, IRF8-PU.1 forms a complex with IRF1 and increases the promoter activity of *IL1β* (70). IRF8 is also required to activate the NLRC inflammasome (71).

IRF8 is involved in polarization of T-cells. In antigen-presenting cells (APCs), IFN-γ binding to IFNGR1/2 transactivates *IRF8* expression through a STAT1-mediated pathway (72). IRF8 binds to the promoter region of *IL12p40* and induces the production of IL-12 p40 from APCs, which promotes differentiation into Th1 cells (73, 74). IL-12p40 is a subunit of IL-12, but it is also a subunit of IL-23, which induces Th17 cell differentiation. In addition, IRF8 regulates *IL27* and *TGFβ* expression in APCs. IRF8 expression decreases IL-27 production and activates TGFβ, thereby enhancing the induction of Th17 cell differentiation (75).

The association between IRF8 and BD has been reported in Chinese populations in addition to that described in the aforementioned Immunochip analysis (76). Functional analysis showed increased *IRF8* mRNA expression and IFN-γ production and decreased production of IL-10 in the risk allele group, indicating higher macrophage differentiation ability in BD (76).

### IFNGR1

A large genetic association study involving 9,444 individuals from seven diverse populations was recently performed and *IFNGR1* and *LNCAROD/DKK1* were identified as new BD susceptibility loci (28). *IFNGR1* expression was increased in CD14^+^ monocytes 2 hours after LPS stimulation in the presence of the risk allele (28). IFNGR1 is a receptor for IFN-γ, and when IFN-γ binds to IFNGR1, it regulates the transcription of genes that contain gamma-activating sequences (GAS) in the promoter region *via* the STAT1 pathway (77). In addition to activating macrophages, IFN-γ has many other functions, including controlling Th cell polarity, enhancing antigen presentation, leukocyte homing and cell adhesion (78). Of note, *IFNGR1* polymorphisms affect the immune response to mycobacterium and *Helicobacter pylori*, which are assumed to be pathogens associated with BD (79, 80). Patients with complete loss of IFNGR1 are repeatedly infected with mycobacterium and develop disseminated Bacille Calmette-Guerin (BCG) and die from BCG inoculation (81, 82). Furthermore, IFNGR1-mediated transactivation of caspase 11 has been reported in mice, indicating that IFNGR1 may be involved in the noncanonical pathway of inflammasome formation (83). These results strengthen the hypothesis of an abnormal innate immune response to external stimuli (including pathogens) in BD pathogenesis. The discovery of the involvement of IGNGR1 and STAT1 pathways may support the use of JAK inhibitors for BD.

## Serum Cytokine Levels in BD

To clarify the role of cytokines, especially those produced by monocytes and macrophages, in BD pathogenesis, we systematically searched PubMed, the Cochrane Central Register of Controlled Trials, and the Web of Science Core Collection (up to November 30, 2021) for literature comparing cytokine levels in healthy controls and BD patients. Search formulae are presented in **Supplementary Text 1**. Differences in cytokine levels between BD and control subjects were calculated as the standardized mean difference (SMD) with confidence intervals (CIs). Pooled analyses were performed using the generic inverse variance method with a random effects model. Heterogeneity was indicated by I^2^, where 0% meant no heterogeneity and 100% meant the strongest heterogeneity. Review Manager version 5.4 software (Cochrane, London, UK) was used to draw paired forest plots.

Among 2,429 candidate articles, we identified 26 eligible studies (84–95). The quality of the original studies was assessed using the Newcastle-Ottawa Quality Assessment Scale for case-control study design (96). Characteristics of the included studies are summarized in **Supplementary Table 1**. The Newcastle-Ottawa Scale score of the included studies was good (**Supplementary Table 2**).

**Figure 4:**
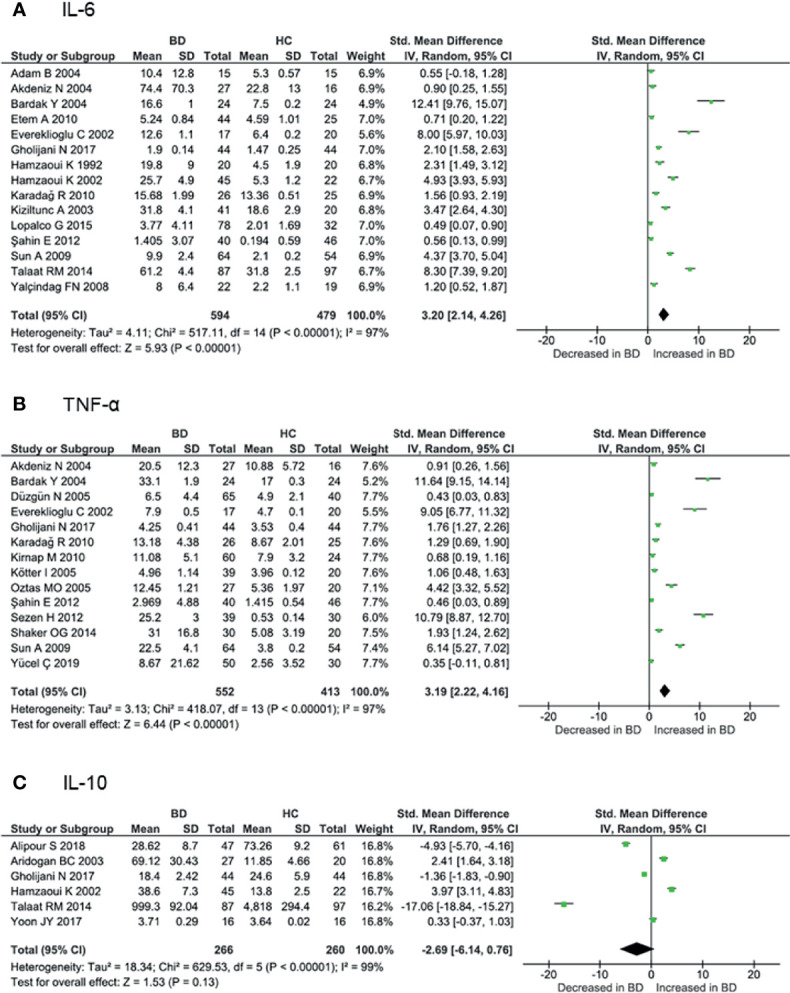
Forest plot of the standardized mean difference of serum cytokine levels in BD patients compared with controls. **(A)** IL-6, **(B)** TNF-α, **(C)** IL-10.

Proinflammatory cytokines play crucial roles in the initiation and perpetuation of disease. Levels of IL-6 and TNF-α are associated with disease activity according to several studies (91, 94). Serum IL-6 levels were measured in 15 cohorts involving 594 BD patients and 479 controls. The meta-analysis showed that IL-6 levels were significantly higher in BD patients than in controls (SMD = 3.20, 95% CI: 2.14–4.26, I^2^ = 97%, p < 0.001) (**Figure 4A**). Similarly, serum TNF-α levels were measured in 14 cohorts involving 552 BD patients and 413 controls, and were significantly increased in BD patients compared with controls (SMD = 3.19, 95% CI: 2.22–4.16, I^2^ = 97%, p < 0.001) (**Figure 4B**). Subgroup analysis revealed that IL-6 and TNF-α levels were increased more in active BD than in inactive BD (**Supplementary Figure 1**). Serum IL-1β levels were studied in only four cohorts involving 191 BD patients and 128 controls. IL-1β levels were increased in BD patients compared with controls (SMD = 1.67, 95% CI: 0.18–3.16, I^2^ = 97%, p < 0.001).

As noted above, IL-10 confers an anti-inflammatory effect involved in the pathogenesis of various autoimmune diseases. Serum IL-10 levels were measured in six cohorts involving 266 BD patients and 260 controls. Some studies have shown that IL-10 is increased in inflamed tissues of BD patients (92, 93). However, serum levels have been reported to be both increased and decreased in BD, and the meta-analysis showed that IL-10 levels were not higher in BD patients compared with controls (SMD = -2.69, 95% CI: -6.14–0.76, I^2^ = 99%, p < 0.001) (**Figure 4C**). Although GWAS have shown decreased *IL10* expression associated with BD (20, 21), various disease activities included in the analyses may have influenced the results (**Supplementary Table 1**). Subgroup analysis revealed that IL-10 levels were higher in active BD than in inactive BD (92, 93).

## Involvement of Monocytes With Current Treatment Strategies

Colchicine inhibits the polymerization of microtubules and is an established treatment for BD. Inhibition of microtubule polymerization prevents NLRP3 from approaching the adopter molecule apoptosis-associated speck-like protein containing a CARD (ASC) and suppresses activation of the NLRP3 inflammasome (97). Colchicine is also associated with increased numbers of nonclassical monocytes, indicating that it may affect monocyte polarization (98). GWAS indicate abnormal inflammasome activation in monocytes, supporting the use of colchicine as an anchor drug for BD.

Anti-TNF-α antibodies have been used to treat uveitis, and intestinal, neurological, and vascular lesions of BD (99). TNF-α receptors include TNFR1 and TNFR2. TNFR1 has the death domain, Tumor necrosis factor receptor type 1-associated DEATH domain (TRADD), and TRADD-mediated aggregation of induced Fas-associated protein with death domain (FADD)and receptor-interacting protein (RIP) activates caspase-8, leading to apoptosis (100). In contrast, TNFR2 does not have a death domain and activation of the TRAF2-mediated pathway leads to the activation of NFκB (101). Haploinsufficiency of A20, encoded by the *TNFAIP3* gene, causes prolonged activation of the NFκB pathway and BD like symptoms (102–105). Anti-TNF antibodies and monocytes have been studied in response to rheumatoid arthritis treatment. Serum monocyte counts are decreased in patients who are responsive to anti-TNF therapy (106) and an early decrease in circulating monocyte count is a predictor of maintenance of remission (107). Anti-TNF antibodies also affect the polarity of macrophages and monocytes. A psoriasis study showed that anti-TNF therapy inhibited the polarity of M1 macrophages in an IRF1- and STAT1-independent manner (108). Another report of inflammatory bowel disease showed that infliximab infusion caused a decrease in monocyte count, which was more pronounced in classical and intermediate monocytes (109).

In 2019, Apremilast produced successful results in a phase 3 trial for refractory oral ulcers in BD (110). Apremilast is an inhibitor of phosphodiesterase (PDE)4, which regulates signal transduction of the intracellular second messengers, cyclic AMP (cAMP) and cyclic GMP (cGMP). The 11 gene families that comprise the PDE superfamily differ in function, primary structure, affinity for cAMP and cGMP, and regulatory mechanisms. Most cells express more than one PDE family member, but the degree of expression varies among tissues and cells (111). PED4 is cAMP-specific and is distributed throughout the body, but is expressed predominantly on T cells, monocytes, macrophages, neutrophils, dendritic cells, and eosinophils (112). *In vivo* stimulation of CD14^+^ monocytes with LPS and apremilast increased the expression of the *SOCS3* gene, which then up-regulated the enhanced IL-10 and IL-6 expression, and decreased IFN-γ expression (113), thus apremilast may correct the genetically driven IL-10 impairment in M2 macrophages of BD patients. Similarly, sub-analysis of a phase 3 trial showed serum IFN-γ levels in the apremilast group were significantly lower than in the placebo group at 12 weeks (114).

## Future Perspectives

Elucidation and interpretation of the function of disease susceptibility loci discovered by GWAS have led to significant progress in understanding the pathogenesis of BD. The pathogenesis of BD cannot be described by an abnormality in a single immune process but is undoubtedly a complex interplay of multiple immune processes. Monocytes/macrophages are responsible for initiating the inflammatory response and directing the subsequent activation of the acquired immune system. Considering that current therapies target monocytes, monocytes are promising targets for new agents, especially those that can alter the polarity of macrophages and monocytes.

The current unmet medical needs of BD are residual disease activity in some patients using current therapies, lack of treatment goals, and personalization of treatment regimens. As seen in the cytokine meta-analysis, the heterogeneity of BD symptoms makes the study of BD difficult. The classification of disease type is necessary to inform therapeutic strategies. We and others have analyzed the phenotypic subtypes of BD and have described five subtypes (115, 116). A disadvantage of categorizing the disease into clinical subtypes is the reduction in patient number for each subtype, especially as BD is a rare disease with a regional bias. A large international cohort to increase the number of participants is, therefore, necessary. In addition to symptom subtypes, there may also be genetic and immunological subsets. IL-17 inhibitors and JAK inhibitors were recently shown to be effective for BD (117, 118). By identifying subsets with predominant monocyte/macrophage activity, subsets with predominant Th17 activity, etc., it may be possible to identify patient groups for which inhibition of specific cells is predicted to be useful. We suggest that further subtype analysis and elucidation of immunological pathogenesis will contribute to personalized medicine in BD.

## Author Contributions

LH, KT-M, and YK designed the research. LH, KT-M, YK, YI-I, YS, and RY conducted the research, statistical analysis, and interpretation of the data. LH, KT-M, and YK drafted the manuscript. YI-I, YS, RY, and HN were involved in writing the article or revising it critically for intellectual content. LH, KT-M, and YK had full access to all the data in the study and take responsibility for the integrity of the data and the accuracy of the data analysis. All authors contributed to the article and approved the submitted version.

## Funding

This study was supported by a Japanese Society for the Promotion of Science Grant-in-Aid for Scientific Research # 19H03700 (YK), and the Japan College of Rheumatology Novartis Pharma Grants for Basic Research 2020. The funder was not involved in the study design, collection, analysis, interpretation of data, the writing of this article or the decision to submit it for publication. All authors declare no other competing interests.

## Conflict of Interest

YK reports personal fees from Amgen, and grant support from Nippon Shinyaku, Tanabe-Mitsubishi, and Chugai.

The authors declare that this study received funding from a Japanese Society for the Promotion of Science Grant-in-Aid for Scientific Research #19H03700 (YK), and the Japan College of Rheumatology Novartis Pharma Grants for Basic Research 2020 (YK). The funder was not involved in the study design, collection, analysis, interpretation of data, the writing of this article or the decision to submit it for publication.

The remaining authors declare that the research was conducted in the absence of any commercial or financial relationships that could be construed as a potential conflict of interest.

## Publisher’s Note

All claims expressed in this article are solely those of the authors and do not necessarily represent those of their affiliated organizations, or those of the publisher, the editors and the reviewers. Any product that may be evaluated in this article, or claim that may be made by its manufacturer, is not guaranteed or endorsed by the publisher.
