# Supplementary material for: The Roles of Monocytes and Macrophages in Behçet’s Disease With Focus on M1 and M2 Polarization
Source: Front Immunol. 2022 Mar 11;13:852297. doi: 10.3389/fimmu.2022.852297 (PMC8963421; doi:10.3389/fimmu.2022.852297)

# *Supplementary Material*

# Supplementary Text 1

# *Search formulas*

**PubMed (n=1,917)**

#1 ((Behcet's) OR (Behçet's) OR (Behcet) OR (Behçet))

#2 ((cytokine) OR (TNF) OR (IL) OR (tumor necrosis factor) OR (tumour necrosis factor) OR (interleukin))

#3 #1 and #2

**Web of Science (n=471)**

#1 TS=((Behcet's) OR (Behçet's) OR (Behcet) OR (Behçet))

#2 TS=((cytokine) OR (TNF) OR (IL) OR (tumor necrosis factor) OR (tumour necrosis factor) OR (interleukin))

#3 #1 and #2

**Cochrane Central Register of Controlled Trials (n=41)**

#1 ((Behcet's) OR (Behçet's) OR (Behcet) OR (Behçet))

#2 ((cytokine) OR (TNF) OR (IL) OR (tumor necrosis factor) OR (tumour necrosis factor) OR (interleukin))

#3 #1 and #2

# Supplementary Tables

**Table 1. Overview of the included studies.**

| **Study** | **Country** | **Demographic data of patients with Behçet disease** | | | **Clinical features of patients with Behçet disease** | | | | | | | | |
| --- | --- | --- | --- | --- | --- | --- | --- | --- | --- | --- | --- | --- | --- |
|  |  | **Total, n** | **Age, years, (Mean ± SD)** | **Female, n (%)** | **Oral ulcers, %** | **Genital ulcers, %** | **Skin lesions, %** | **Ocular, %** | **Articular, %** | **Vascular, %** | **Neurological, %** | **Gastro-intestinal, %** | **Active patients, n (%)** |
| **Adam B 2004** | Turkey | 15 | 33  (Range 21–52) | 6 (40.0%) | NR | NR | NR | NR | NR | NR | NR | NR | 15 (100%)^a^ |
| **Akdeniz N 2004** | Turkey | 27 | 26±5 | 15 (55.6%) | 100% | 100% | 55.3% | 63.7% | 100%^b^ | 18.8%^c^ | 8.2% | 4.1% | NR |
| **Alipour S 2018** | Iran | 47 | 38±10 | 18 (38.2%) | 96% | 49% | 84.5%^d^ | 77% | 19% | NR | NR | NR | 30 (63.8%)^e^ |
| **Aridogan BC 2003** | Turkey | 27 | 34±11 | 15 (55.6%) | NR | NR | NR | NR | NR | NR | NR | NR | 27 (100%)^f^ |
| **Bardak Y 2004** | Turkey | 24 | 28±6 | 7 (29.2%) | NR | NR | NR | NR | NR | NR | NR | NR | 24 (100%)^g^ |
| **Düzgün N 2005** | Turkey | 65 | NR | NR | 100% | 43.1% | 55.4% | 23.1% | NR | 13.8% | 4.6% | NR | 65 (100%)^h^ |
| **Etem A 2010** | Turkey | 44 | 36±11 | 25 (56.9%) | 50% | 27.3% | 20.5% | 25.0% | 40.9% | NR | 20.5% | NR | 21 (47.7%)^i^ |
| **Evereklioglu C 2002** | Turkey | 37 | 37 | 18 (48.6%) | 100% | 83.7% | 91.8% | 51.3% | 89.1% | NR | 24.3% | 21.6% | 17 (45.9%)^j^ |
| **Gholijani N 2017** | Iran | 44 | 38±9 | 21 (47.7%) | NR | NR | NR | 13.6% | NR | NR | NR | NR | 29 (65.9%)^k^ |
| **Hamzaoui K 1992** | Tunisia | 20 | 36±7 | 2 (10.0%) | 95% | 90% | NR | 95% | 70% | NR | NR | NR | 20 (100%) |
| **Hamzaoui K 2002** | Tunisia | 45 | NR | 0 (0%) | NR | NR | NR | NR | NR | NR | NR | NR | 25 (55.6%)^l^ |
| **Karadağ R 2010** | Turkey | 26 | 36±8 | 19 (73.1%) | NR | NR | NR | NR | NR | NR | NR | NR | 26 (100%) |
| **Kirnap M 2010** | Turkey | 60 | 36±9 | 36 (60.0%) | NR | NR | NR | NR | NR | NR | NR | NR | NR |
| **Kiziltunc A 2003** | Turkey | 41 | 34±8 | 19 (46.3%) | NR | NR | NR | NR | NR | NR | NR | NR | 19 (46.3%)^m^ |
| **Kötter I 2005** | Germany | 50 | NR | 14 (28.0%) | NR | NR | NR | 100% | NR | NR | NR | NR | 100% |
| **Lopalco G 2015** | Italy | 58 | 45±12 | 30 (51.7%) | 39.7% | 12.1% | 29.3% | 31.0% | NR | 5.2% | 10.3% | 24.1% | NR |
| **Oztas MO 2005** | Turkey | 27 | 34±12 | 9 (33.3%) | 100% | 81.5% | 96.2% | 25.9% | NR | NR | NR | NR | 100% |
| **Şahin E 2012** | Turkey | 40 | 38±11 | 18 (45.0%) | 100% | 97.5% | 45.0%^n^ | 20.0% | NR | NR | NR | NR | 31 (77.5%)^o^ |
| **Sezen H 2012** | Turkey | 39 | NR | NR | NR | NR | NR | NR | NR | NR | NR | NR | 39  (100%) |
| **Shaker OG 2014** | Egypt | 30 | 34±10 | 0 (0%) | 63.3% | 46.7% | 16.7%^p^ | 40.0% | NR | 20.0% | NR | NR | NR |
| **Sun A 2009** | Taiwan | 64 | 42  (Range 11–73) | 41 (64.1%) | 100% | 100% | NR | NR | NR | NR | NR | NR | NR |
| **Talaat RM 2014** | Egypt | 87 | 34±10 | 16 (18.4%) | 100% | 92% | 54% | 63.2% | 31% | 28.7% | 21.8% | 5.7% | 37 (42.5%)^q^ |
| **Yalçindag FN 2008** | Turkey | 22 | 33±8 | 10 (45.5%) | 100% | NR | NR | 63.6% | NR | NR | NR | NR | NR |
| **Yoon JY 2017** | Korea | 16 | 50±9 | 11 (68.8%) | 100% | 87.5% | 93.8% | 62.5% | 31.3% | 37.5% ^r^ | NR | NR | NR |
| **Yosipovitch G 1995** | Israel | 25 | 38  (Range 18-62) | 16 (64.0%) | NR | NR | NR | NR | NR | NR | NR | NR | NR |
| **Yücel Ç 2019** | Turkey | 50 | 39±9 | 17 (34.0%) | 100% | 78.0% | 48.0% ^s^ | 40.0% | 42.0% | 44.0% | NR | 2.0% | 27 (54.0%)^t^ |

Abbreviations: NR = not reported.

^a^ Mucocutaneous disease or involvement of various organs. ^b^ Arthralgia. ^c^ Venous disease. ^d^ Erythema nodosum. ^e^ Sever Behçet disease. ^f^ Oral and/or genital ulcerations, pathergy test positivity, eye lesions, and arthritis/arthralgia were examined and given colchicine treatment. ^g^ Active uveitis. ^h^ At least two of the following were considered to have active disease: oral ulcers, genital ulcers, ocular involvement, cutaneous lesions typical of Behçet disease, arthritis, vascular lesions, and pulmonary and central nervous system manifestations. ^i^ At least three of the major symptoms at the time of study (oral ulcers, genital ulcers, skin lesions, uveitis, positive pathergy test) were considered to indicate the active period of the disease. ^j^ In clinical evaluation, worsening of clinical symptoms at the time of study and having at least three of the major symptoms (oral ulcers, genital ulcers, skin lesions and uveitis) were considered to be in the active period of the disease. ^k^ At least three of the main Behçet disease symptoms. oral ulcers, genital ulcers, uveitis, skin lesion, arthritis. ^l^ Oral ulcers, genital ulcers, uveitis, skin lesion, arthritis. ^m^ Patients who had three of the five findings (genital ulceration, positive pathergy test, oral ulceration, skin lesions and eye lesions), or who had both skin lesions and ocular lesions, or who had multiple erythema nodosum with severe inflammation and with both elevated ESR and positive CRP were assumed to have active disease. ^n^ Erythema nodosum. ^o^ At least three of the following clinical features were included in the active group: oral aphthae, genital ulcer, uveitis, skin lesions and positive pathergy response. ^p^ Erythema nodosum. ^q^ Patients with two or more lesions in the previous 4 weeks (including oral ulcers, genital ulcers, skin lesions, uveitis, vasculaities, arthritis, gastrointestinal lesions, CNS lesions, and pulmonary involvement) were regarded to have active disease. ^r^ Vasculitis. ^s^ Erythema nodosum. ^t^ Disease activity was assessed with the Turkish version of the BD Current Activity Form (BDCAF). The items of this activity include headache, oral ulcers, genital ulcers, erythema, skin pustules, arthralgia, arthritis, intestinal involvement, new eye involvement, new nervous system involvement, and vascular involvement (venous thrombosis and/or arterial aneurysms).

**Table 2. The Newcastle-Ottawa Scale for case-control studies.**

|  | **Selection** | | | | **Comparability** | **Exposure** | | |  |
| --- | --- | --- | --- | --- | --- | --- | --- | --- | --- |
| **Author and Year** | Is the Case Definition Adequate? | Representativeness of the Cases | Selection of Controls | Definition of Controls | Comparability of Cases and Controls on the Basis of the Design or Analysis | Ascertainment of Exposure | Same Method of Ascertainment for Cases and Controls | Non-Response Rate | **Total** |
| **Adam B 2004** | * | * | * | * | * |  | * | * | **7** |
| **Akdeniz N 2004** | * | * | * | * | * |  | * | * | **7** |
| **Alipour S 2018** | * | * | * | * | * |  | * | * | **7** |
| **Aridogan BC 2003** | * | * | * | * | * |  | * | * | **7** |
| **Bardak Y 2004** | * | * | * | * | * |  | * | * | **7** |
| **Düzgün N 2005** | * | * | * | * | * |  | * | * | **7** |
| **Etem A 2010** | * | * | * | * | * |  | * | * | **7** |
| **Evereklioglu C 2002** | * | * | * | * | * | * | * | * | **8** |
| **Gholijani N 2017** | * | * | * | * | * |  | * | * | **7** |
| **Hamzaoui K 1992** | * | * | * | * | * |  | * | * | **7** |
| **Hamzaoui K 2002** | * | * | * | * | * |  | * | * | **7** |
| **Karadağ R 2010** | * | * | * | * | * |  | * | * | **7** |
| **Kirnap M 2010** | * |  | * | * | * |  | * | * | **6** |
| **Kiziltunc A 2003** | * | * | * | * | * |  | * | * | **7** |
| **Kötter I 2005** | * |  | * | * | * |  | * |  | **5** |
| **Lopalco G 2015** | * |  | * | * | * |  | * |  | **5** |
| **Oztas MO 2005** | * | * | * | * | * |  | * | * | **7** |
| **Şahin E 2012** | * | * | * | * | * |  | * | * | **7** |
| **Sezen H 2012** | * | * | * | * | * |  | * | * | **7** |
| **Shaker OG 2014** | * | * | * | * | * |  | * | * | **7** |
| **Sun A 2009** | * | * | * | * | * |  | * | * | **7** |
| **Talaat RM 2014** | * | * | * | * | * |  | * | * | **7** |
| **Yalçindag FN 2008** | * | * | * | * | * |  | * | * | **7** |
| **Yoon JY 2017** | * | * | * | * | * |  | * | * | **7** |
| **Yosipovitch G 1995** | * | * | * | * | * |  | * | * | **7** |
| **Yücel Ç 2019** | * | * | * | * | * |  | * | * | **7** |

# Supplementary Figures

Figure 1. Forest plot of the standardized mean difference of serum cytokine levels in active patients compared with inactive patients.

1. IL-6


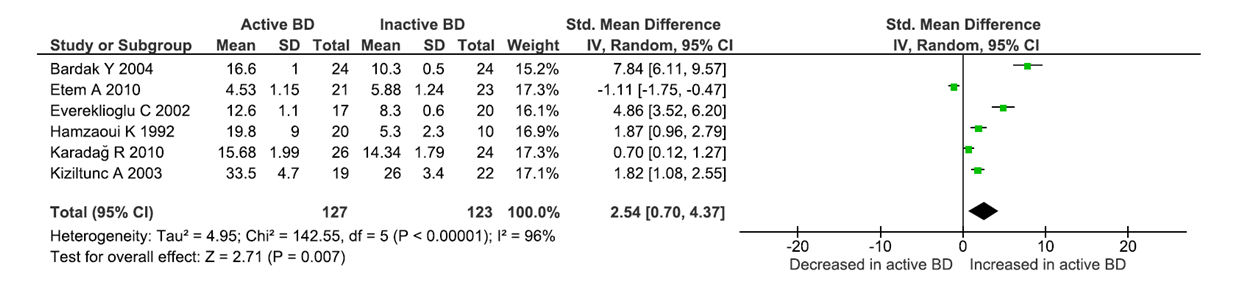


1. TNF-α


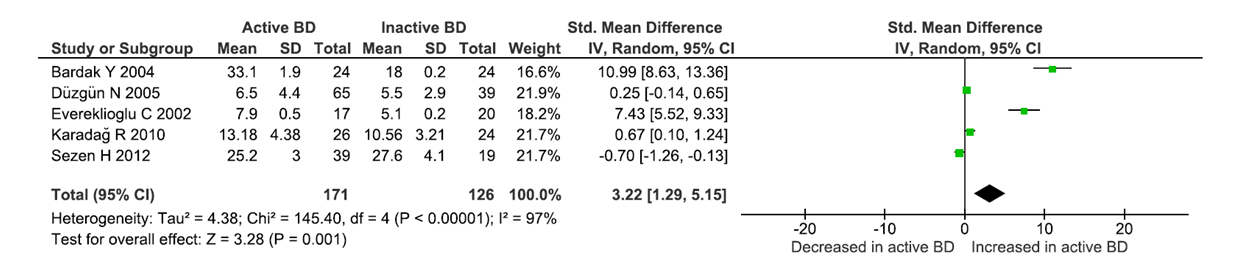


Figure 2. **A**; single cell mRNA expression analysis of CCR1 in various leukocytes. CCR1 is highly expressed in low density granulocytes (LDG), neutrophils (Neu), and classical monocytes (CL_Mono). **B and C**; expression quantitative expression analysis of CCR1 and CCR3 of Behçet’s disease-susceptible SNP rs7616215. Allele T is associated with a risk of Behçet’s disease. Note that T allele is associated with reduced expression of CCR1 in plasmacytoid dendritic cells (pDC), Neu, CL_Mono, and other leukocytes. Figure adapted from a publication by Ota et al, *Cell*. 2021; 184(11): 3006-3021. Figure downloaded from ImmuNexUT (<https://www.immunexut.org/>). Distributed under the Creative Commons Attribution 4.0 International License (CCBY4.0, http://creativecommons.org/licenses/by/4.0/).


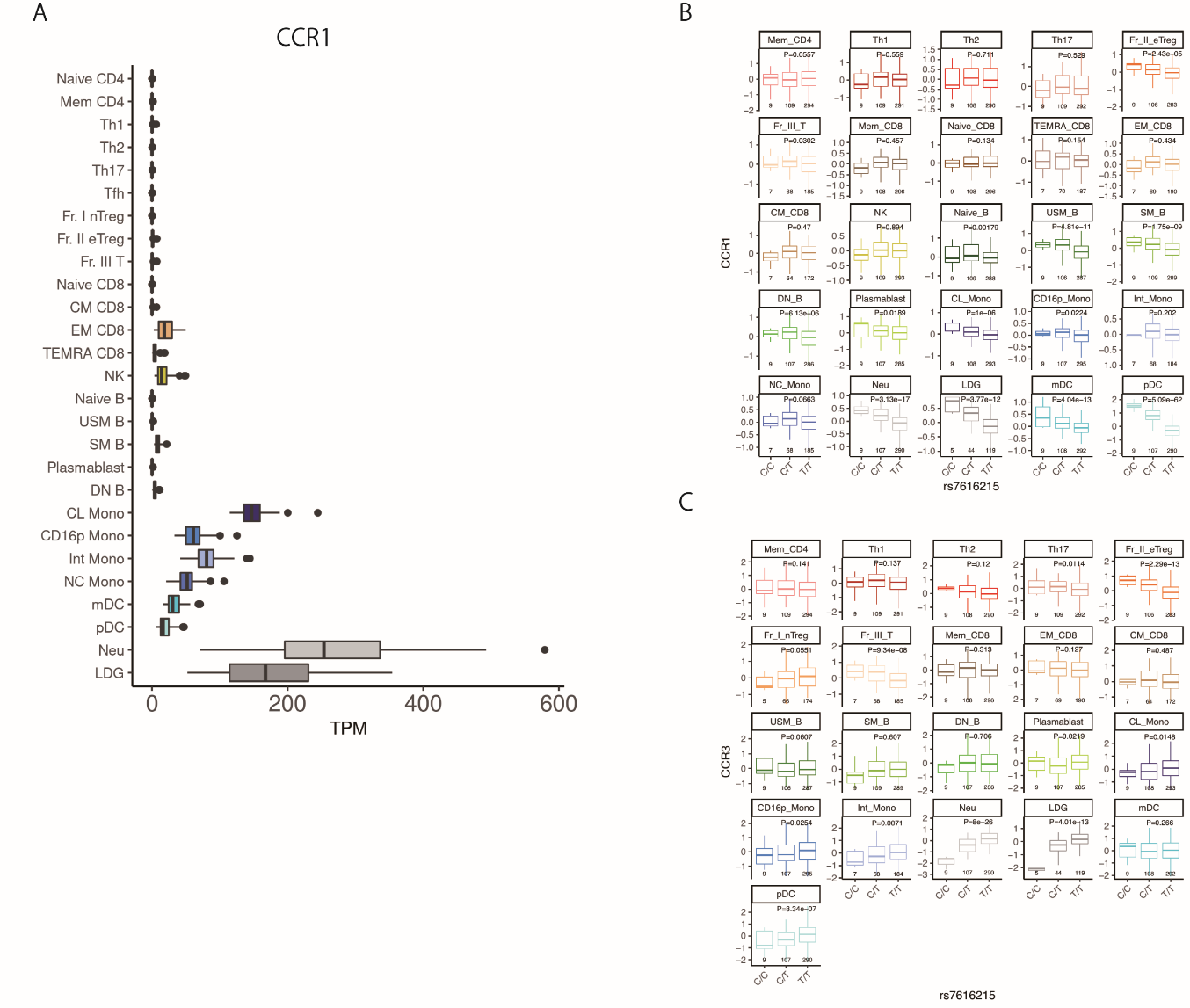

Supplement: Supplementary file 1 [file DataSheet_1.docx]
